# Room at the top

**DOI:** 10.7554/eLife.31697

**Published:** 2017-10-12

**Authors:** Randy Schekman

**Keywords:** scientiific publishing, peer review, eLife, research assessment

## Abstract

Five years after eLife published its first papers, we reflect on our consultative approach to peer review, the challenges of reproducibility, and the need to reform how published research is assessed.

From the outset, our goal at eLife has been to build a selective, open-access journal where the work of researchers in the life and biomedical sciences would be evaluated by other active researchers. Moreover, funded by the Howard Hughes Medical Institute, the Max Planck Society and the Wellcome Trust, we wanted to make the peer-review process itself fairer and more constructive. We also wanted to start a debate about how published research was assessed, which meant eschewing the use of the journal impact factor as a proxy for the importance or significance of a particular paper. Our position is that we are not in the business of selling magazines and that we publish papers because our editors think they contain science that is both rigorous and outstanding, not because we think they will attract lots of citations and improve our impact factor.

In the years before eLife was launched it had become clear that there was an appetite among researchers for an alternative to the sometimes combative style of peer review that appeared to be the norm at the most selective journals. Part of the problem, we felt, was that reviewers remained anonymous to each other and to the author, so we decided to use a consultative approach in which each reviewer knew who the other reviewers were and, once the final review had been submitted, the reviewers could discuss their thoughts on the manuscript and the other reviews. Our aim was to create a forum for reviewers to judge work together as peers, as happens when research is presented at scientific meetings or when grants are reviewed by panels or study groups.

For those manuscripts where the reviews are favorable, the Reviewing Editor drafts a decision letter that contains a consolidated list of the revisions the authors will need to make in order to have their manuscript accepted for publication. We feel that this consultative approach to peer review has worked well: in particular, when there is a difference of opinion among the reviewers, the consultation process often results in a measured and constructive resolution of these differences. The published version of the paper includes the decision letter and the authors' response to it. Several other journals have adopted all or parts of our consultative review process (King, 2017).

Although the consultation requires a bit more work than the traditional approach to peer review, many reviewers feel that they are taking a more active role in reaching a decision about the manuscript. Many of our new reviewers find this approach to be refreshing and they often wonder why it has not been adopted more widely. We also find that students appreciate the insights into the peer-review process provided by the publication of the decision letter and the author response. Our efforts to make the work published in eLife of interest to the wider research community (via Insights) and the general public (via plain-language summaries called digests) have also been well received.

We are also working with the Center for Open Science (COS) and Science Exchange on an initiative to independently replicate selected results from a number of high-profile papers in the field of cancer biology. The Reproducibility Project: Cancer Biology was prompted by reports that drug companies had not been able to reproduce the results of a large fraction of highly-cited cancer biology papers. So far we have published 29 Registered Reports and 7 Replication Studies as part of this project. To date the majority of the Replication Studies have reproduced important parts of the original paper, but one has not and two could not be interpreted. The bottom line is that there will be no certain answers but the project has given us valuable insights into the difficulties of reproducing complex biological results with fixed resources and a rigid adherence to the published protocols. And to improve reproducibility more generally we have started to publish the 'transparent reporting forms' in which authors provide information about sample sizes, replicates, statistical reporting and source data (Teare, 2016).

There is room for an alternative where active researchers make all the key decisions about manuscripts and share their views in a constructive review process

One feature of the past five years has been growth -- in submissions (some 5,766 in the first nine months of this year), research papers published (983 for the same period), and the number of Senior Editors and Reviewing Editors making decisions for the journal (currently 40 and 329 respectively). It is particularly important to note that, as an online journal, eLife does not have a page budget: this means that we are able to publish all the articles that reach the standards set by our editors. One consequence of growth is that we decided to implement a publication fee of $2500 per article at the start of 2017 (Schekman and Patterson, 2016). This fee is in no way intended to cover all our costs: rather, it is the marginal cost we incur for publishing an article. It does not, for example, cover any of the costs involved in the development of the open-source publishing software we have developed, such as eLife Lens (which allows the text and figures in papers to be viewed side-by-side) or eLife Continuum (our journal hosting platform).

Regarding the need to rethink how published research is evaluated by funding agencies, academic institutions and other parties, we have embraced the Declaration on Research Assessment (DORA). Three overarching themes run through the DORA recommendations: the need to eliminate the use of journal-based metrics, such as journal impact factors, in decisions about funding, appointments and promotions; the need to assess research on its own merits, not the journal in which it is published; and the need to take full advantage of the opportunities offered by online publication (by, for example, exploring new indicators of significance and impact, and not placing unnecessary limits on the length of articles). It is comforting to note that the American Association for the Advancement of Science, which publishes Science, was one of the original signatories of DORA, and that Nature Research recently signed the declaration.

At this point I measure success not by some crude metric, but by the support of our editorial board and the authors who send us their outstanding work. Our goal is not to supplant any of the traditional 'high-impact' journals that are so dominant in the life and biomedical sciences: rather I believe there is room at the top for an alternative where active researchers make all the key decisions about manuscripts and share their views in a constructive review process. If you have not already experienced this approach, please give us a try!
